# Prognostic significance of serum galectin-3 in predicting cardiovascular outcomes after percutaneous coronary intervention with drug-eluting stents

**DOI:** 10.3389/fcvm.2025.1563068

**Published:** 2025-07-17

**Authors:** Yeon-Jik Choi, Suk Min Seo

**Affiliations:** Department of Internal Medicine, Cardiovascular Center and Cardiology Division, Eunpyeong St. Mary’s Hospital, College of Medicine, The Catholic University of Korea, Seoul, Republic of Korea

**Keywords:** galectin-3, predictor, coronary artery disease, percutaneous coronary intervention, drug-eluting stents

## Abstract

**Background:**

Galectin-3 is a well-established biomarker on the predictor of cardiovascular events in patients with heart failure. Its pathophysiologic association with inflammation, cell proliferation, and fibrogenesis may implicate serum galectin-3 as a predictor of clinical outcomes in coronary artery disease (CAD) patients undergoing percutaneous coronary intervention (PCI) with drug-eluting stents (DES). The aim of this study was to examine the prognostic value of the galectin-3 level in patients with CAD who underwent PCI with DES.

**Methods:**

A total of 939 patients undergoing successful PCI with DES were consecutively enrolled between January 2007 and December 2009. The serum galectin-3 level was measured, classified into two groups according to the median galectin-3 level (9.52 ng/ml, interquartile range 7.31–12.81), and compared with the composite of all-cause mortality, non-fatal myocardial infarction (MI), and stroke.

**Results:**

The median follow-up duration was 997 days (interquartile range 766–1,264 days). The high galectin-3 group had a significantly higher incidence of all-cause mortality, cardiac mortality, and composite of all-cause mortality, non-fatal MI, and stroke. High galectin-3 was a significant independent predictor of the composite of all-cause mortality, non-fatal MI, and stroke (adjusted hazard ratio 1.670, 95% confidence interval 1.014–2.751, *p* = 0.044). The addition of the serum galectin-3 level to the conventional clinical risk model improves the model discrimination (C-statistic = 0.694–0.786, *p* for difference < 0.01), reclassification [continuous net classification improvement (0.297, *p* < 0.01) and integrated discrimination improvement (0.064, *p* < 0.01)].

**Conclusion:**

Our data suggest that serum galectin-3 is an independent predictor of cardiovascular events in patients undergoing PCI with DES.

## Introduction

Inflammation involves the entire process of atherosclerosis, foam cell formation, plaque progression and rupture, and thrombus formation (1). Galectin-3 is a member of a family of proteins comprising soluble β-galactoside-binding lectins that have regulatory roles in fibrogenesis, inflammation, tissue repair, and cell proliferation (2). Galectin-3 plays an active part in the atherogenic process, and it induces the transformation of macrophage into foam cells. Highly upregulated galectin-3 is expressed when monocytes differentiate into macrophages (3), and also when macrophage or aortic smooth muscle cells are loaded with lipids and transformed into foam cells (4, 5). Plaque foam cells may secrete galectin-3, and it plays a potent chemoattractant for monocytes and macrophages, thus enhancing its recruitment from the peripheral blood to the artery wall (6).

Clinically, increased inflammation activity of the vessel wall is strongly associated with cardio-cerebral events. While the C-reactive protein (CRP) is assumed to be a good biomarker for evaluating the severity of inflammation of the vessel wall, it has conflicting results as a prognostic predictor in patients with myocardial infarction (MI): positively correlated in some studies (7, 8) but little correlated with prognosis in other studies (9–11). Since the routine usage of CRP as a prognostic factor of coronary artery disease (CAD) is not clear, it is still an unmet clinical need to find prognostic predictors in high-risk patients with CAD. While serum galectin-3 levels are an important risk factor in patients with heart failure, their prognostic implications are unknown in patients with CAD. In this study, we investigated whether the initial serum galecctin-3 levels could predict outcomes in patients undergoing percutaneous coronary intervention (PCI) with drug-eluting stents (DES).

## Methods

### Study subjects

The analysis was carried out from a single-center registry of CAD patients undergoing successful PCI with DES at Seoul St. Mary's Hospital. We consecutively enrolled patients who provided informed consent for enrollment in the registry and blood bank. Patients were included regardless of the clinical presentation [stable angina or acute coronary syndrome (ACS)] at the time of the PCI. We analyzed the serum galectin-3 level of 939 patients between January 2007 and December 2009. There was no involvement of the industry in the design, conduct, or analysis of the study. The study protocol was reviewed and approved by the institutional review boards.

### PCI and medical treatment

Before the PCI, all patients received a daily dose of300 mg of aspirin. Clopidogrel with a 600 mg loading dose was administered at least 1 day before the procedure. The procedure was performed through the femoral or the radial artery after the administration of unfractionated heparin (100 U/kg). During the procedure, patients received unfractionated heparin to maintain an activated clotting time between 250 and 300 s. The choice of the stent was at each physician's discretion and the stent was deployed after balloon angioplasty. A successful PCI was defined as a decrease in the minimum stenosis diameter to less than 30% with thrombolysis in myocardial infarction grade III flow on coronary angiogram. During the in-hospital period, dual antiplatelet and statin were administered to all patients. After discharge, the patients continued receiving the same medications, except for some intravenous or temporary medications.

### Assessment of laboratory data

Fasting blood samples were obtained within 24 h prior to the PCI and were used to perform the standard battery of hematological and biochemical tests. These samples were used to measure high-sensitivity C-reactive protein (hs-CRP) using the high-sensitivity immunoturbidimetric method. In the case of emergency patients, the samples were used at the time of entry into the emergency room.

Blood sampling for the measurement of serum galectin-3 was done at the catheterization laboratory room before the PCI. The blood sample was stored by arteriopuncture prior to the PCI regardless of fasting in ethilen dianmin acetic acid (EDTA)-containing tubes. After centrifugation, plasma samples were stored at −80℃ in a refrigerator. The serum galectin-3 level was measured by a novel and optimized enzyme-linked immunosorbent assay method using the human Galectin-3 Quantikine kit (R&D Systems, Inc., Minneapolis, MN, USA). The measuring range provided by the manufacturer, which is defined by the lower detection limit, is 0.016 ng/mL. The measurement of serum galectin-3 was performed by the Seoul Medical Science Institute, a fiduciary institution that professionally examines clinical specimens.

### Study end point and definition

The records of cardiovascular risk factors, history, and laboratory findings were mainly obtained from the electronic medical records of the patients. All-cause mortality was considered to be cardiac mortality after the exclusion of non-cardiac mortality. MI was defined as myocardial damage detected by abnormal cardiac biomarkers in acute myocardial ischemia, which corresponds to the universal definition of acute myocardial infarction. Unstable angina was defined as new-onset chest pain, angina at rest, and angina of increasing frequency or intensity. Target lesion revascularization (TLR) was defined as ischemia-induced PCI of the target lesion resulting from restenosis or re-occlusion within the stent or in the adjacent 5 mm of the distal or proximal segments. Target vessel revascularization (TVR) was also defined as any segment of the epicardial coronary artery containing the target lesion (12).

The primary objective of the present study was to evaluate the association between galectin-3 and the composite of all-cause mortality, non-fatal MI, and stroke. The secondary objective was to evaluate whether a higher galectin-3 level would be associated with all-cause mortality, non-fatal MI, stroke, revascularization, and major adverse cardiac events (MACE) including all-cause mortality, non-fatal MI, stroke, and any revascularization. MI includes ST segment elevation MI (STEMI) and non-STEMI (NSTEMI) excluding periprocedural MI. Stroke, as indicated by neurologic deficits, was confirmed by a neurologist on the basis of imaging studies. Any revascularization includes TVR and non-TVR with the use of either PCI or coronary artery bypass graft (CABG).

The clinical, angiographic, procedural or operative, and outcome data were collected from the dedicated PCI and surgical databases by independent research personnel. For the validation of complete follow-up data, information on censored survival data was obtained through 31 December 2011 from a telephonic interview with the corresponding patients and also from the database of the National Health Insurance Corporation, Korea, using a unique personal identification number.

### Statistical analysis

Continuous variables were expressed as mean ± standard deviation and were compared with Student's t-test or the Mann–Whitney *U* test. Discrete variables were expressed as percentages and compared using the *χ^2^* test or Fisher's exact test. A multivariable Cox regression analysis (after confirming the appropriateness of the proportional hazards assumption) was conducted to identify independent predictors for cardiovascular events. A univariate Cox regression analysis was conducted with variables with a statistical *p*-value of <0.1 in the baseline characteristics (Table 1). Then, variables with a significant association (*p* < 0.05) in the univariate analysis were evaluated in the multivariable Cox regression model. The effect of each variable in the developing models was assessed using the Wald test and described as hazard ratios (HRs) with 95% confidence intervals (CIs). The cumulative survival rate was estimated by using the Kaplan–Meier survival curves and compared using the log-rank tests. A receiver operating characteristic (ROC) curve analysis using Youden index J analyses was performed to find the optimal cutoff value of galectin-3 and hs-CRP with the highest sensitivity and specificity associated with the occurrence of events. We performed Cox regression analysis after dividing the patients into four groups according to the cutoff values of galectin-3 and hs-CRP by ROC curves. This Cox regression analysis used two models: (i) model 1: conventional clinical risk model with prognostic impact shown in previous studies included age, male gender, diabetes, hypertension, smoking, and hypercholesterolemia; (ii) model 2: model 1 variables and additional covariates with a significant association (*p* < 0.05) in the univariate analysis such as initial presentation of acute MI (AMI), C-reactive protein, glucose, estimated glomerular filtration rate (eGFR), left ventricular ejection fraction, troponin-T, triglyceride, and lesion extent.

**Table 1:** Baseline demographic, clinical, and angiographic data according to galectin-3 level.

Variables	Low Gal-3 (*n* = 469)	High Gal-3 (*n* = 470)	*p*-value
Clinical characteristics			
Age (years)	60.7 ± 10.0	65.0 ± 10.8	<0.0001
Age ≥65 years	178 (38.0)	250 (53.2)	<0.0001
Male gender	362 (77.2)	299 (63.6)	<0.0001
Risk factors			
BMI (kg/m^2^)	24.4 ± 3.9	24.1 ± 4.8	0.188
Diabetes mellitus	154 (32.8)	225 (47.9)	<0.001
Hypertension	267 (56.9)	301 (64.0)	0.026
Current smoking	118 (25.2)	96 (20.4)	0.084
Family history of CAD	40 (8.5)	36 (7.7)	0.625
Prior history of stroke			
Prior history of MI	26 (5.5)	35 (7.4)	0.237
Prior history of PCI	48 (10.2)	55 (11.7)	0.472
Prior history of CABG	7 (1.5)	5 (1.1)	0.559
Initial vital sign			
Systolic blood pressure (mmHg)	127.3 ± 29.6	128.4 ± 29.0	0.715
Diastolic blood pressure (mmHg)	78.0 ± 18.7	78.4 ± 17.2	0.833
Heart rate	77.6 ± 20.4	76.1 ± 19.3	0.465
Primary diagnosis at admission			<0.001
Stable angina	240 (51.2)	170 (36.2)	
Unstable angina	101 (21.5)	106 (22.6)	
NSTEMI	38 (8.1)	72 (15.3)	
STEMI	65 (13.9)	98 (20.9)	
Silent ischemia	25 (5.3)	24 (5.1)	
AMI	103 (22.0)	170 (36.2)	<0.001
Medication prior PCI			
Aspirin	193/404 (47.8)	196/378 (51.9)	0.254
Statin	107/404 (26.5)	98/378 (25.9)	0.859
Discharge medication			
Aspirin	463/467 (99.1)	457/458 (99.8)	0.186
Clopidogrel	469/469 (100)	469/470 (99.8)	0.318
Statin	411/466 (88.2)	398/458 (86.9)	0.550
Beta-blocker	347/466 (74.5)	360/458 (78.6)	0.138
ACEI or ARB	395/466 (84.8)	393/458 (85.8)	0.654
CCB	107/466 (23.0)	105/458 (22.9)	0.990
Laboratory data			
LVEF (%)	57.5 ± 16.9	54.2 ± 18.0	0.003
Glucose (mg/dL)	118.2 ± 41.1	128.8 ± 59.5	0.002
Creatinine (mg/dL)	1.01 ± 0.4	1.53 ± 2.0	<0.001
eGFR (mL/min/1.73 m^2^)	71.2 ± 16.8	62.2 ± 23.0	<0.001
Reduced kidney function (eGFR < 60)	106 (22.6)	192 (40.9)	<0.001
Hs-CRP (mg/L)	0.53 ± 1.54	1.39 ± 3.70	<0.001
CK-MB (ng/mL)	8.8 ± 32.4	14.4 ± 42.9	0.024
Troponin-T (ng/mL)	3.12 ± 10.6	5.42 ± 13.03	0.003
Total cholesterol (mg/dL)	173.4 ± 40.1	165.6 ± 47.1	0.006
Triglyceride (mg/dL)	141.4 ± 122.8	129.0 ± 81.5	0.069
HDL-cholesterol (mg/dL)	43.1 ± 12.0	42.3 ± 13.6	0.340
LDL-cholesterol (mg/dL)	103.8 ± 35.9	97.6 ± 39.7	0.012
Hypercholesterolemia	109 (23.2)	100 (21.3)	0.469
Lipoprotein (g/mL)	24.6 ± 23.7	25.0 ± 24.2	0.787
Angiographic data			
Left main disease	24 (5.1)	18 (3.8)	0.340
Disease vessels, *n* (%)			<0.001
1 vessel	233 (49.7)	177 (37.7)	
2 vessels	146 (31.1)	154 (32.8)	
3 vessels	90 (19.2)	139 (29.6)	
Lesion characteristics, *n* (%)			0.135
A/B1	78 (16.6)	93 (20.4)	
B2/C	391 (83.4)	374 (79.6)	
Stent number per patient	1.15 ± 0.54	1.18 ± 0.57	0.419
Mean stent diameter (mm)	3.23 ± 0.43	3.13 ± 0.44	<0.001
Total stent length (mm)	28.16 ± 15.22	29.38 ± 16.24	0.235

ACEI/ARB, angiotensin converting enzyme inhibitor/angiotensin II receptor blocker; AMI, acute myocardial infarction; B2/C, complex lesion; BMI, body mass index; CABG, coronary artery bypass graft; CAD, coronary artery disease; CCB, calcium channel blocker; CK-MB, creatine kinase-MB fraction; eGFR, estimated glomerular filtration rate; HDL, high-density lipoprotein; Hs-CRP, high-sensitivity C-reactive protein; LDL, low-density lipoprotein; LVEF, left ventricular ejection; NSTEMI, non-ST segment elevation myocardial infarction; PCI, percutaneous coronary intervention; STEMI, ST segment elevation myocardial infarction.

Data are presented as mean ± SD or *n* (%).

We wanted to assess the incremental prognostic value of the addition of biomarkers including galectin-3 and hs-CRP to model 1: conventional clinical risk. We performed several sensitivity analyses that confirmed the consistency of the models. The discriminatory abilities of the models were assessed using the C-statistic, and differences in the C-statistic between the models were compared according to the method of DeLong et al. (13). The calibration of the two models was evaluated using the Hosmer–Lemeshow goodness-of-fit test, and model fitness was assessed using the Akaike information criterion. Furthermore, to estimate whether the addition of biomarkers to the conventional clinical risk model led to any significant risk reclassification of all-cause mortality, net reclassification index (NRI), and integrated discrimination improvement (IDI) were calculated according to the method described by Pencina et al. (14). All statistical analyses were two-tailed, with clinical significance defined as values of *p* <0.05. All statistical analyses were conducted using the Statistical Analysis Software package (SAS version 9.1, SAS Institute, Cary, NC, USA) and R (version 3.3.2).

## Results

### Characteristics of the study population

Serum galectin-3 levels ranged from 2.44 to 90.40 ng/mL, had a mean value of 11.68 ± 8.25 ng/mL, and a median of 9.52 ng/mL (interquartile range 7.31–12.81). All patients were divided into two groups according to the median galectin-3 level: high galectin-3 group (*n* = 470) and low galectin-3 group (*n* = 469).

Baseline demographic, clinical, laboratory, and angiographic characteristics between the two groups are given in Table 1. Patients in the high galectin-3 group were older, with the female gender predominating, and had a higher prevalence of diabetes, hypertension, and presentation of AMI. These patients with high galectin-3 were more likely to have a higher hs-CRP, higher cardiac enzyme levels, lower left ventricular ejection fraction, lower LDL-cholesterol, and a lower eGFR. Patients with high galectin-3 had higher incidences of three-vessel disease and less mean stent diameter on coronary angiogram. There were no differences in medications between the two groups. Baseline characteristics according to whether ACS is present or not, are shown in Table S1.

### Clinical outcomes for the study populations

The median follow-up duration was 997 days (interquartile range, 766–1,264). Follow-up data for cardiac events were obtained in 100% of the overall cohort for the duration of this study. The follow-up coronary angiogram was performed in 67.2% (*n* = 631) of the overall cohort at a median follow-up of 394 days (interquartile range, 300–795).

In the high galectin-3 group, the composite of all-cause mortality, non-fatal MI, and stroke occurred in a total of 65 patients (13.8%), while in the low galectin-3 group, they occurred only in 27 patients (5.8%) during long-term follow-up. The incidence of all-cause mortality, cardiac mortality, composite of cardiac mortality, non-fatal MI, and stroke, and composite of MACE was significantly higher in patients with high galectin-3 than in those with low galectin-3 (Table 2).

**Table 2:** Cumulative clinical outcomes.

Variables	Low Gal-3 (*n* = 469)	High Gal-3 (*n* = 470)	*p*-value
All-cause death	20 (4.3)	52 (11.1)	**<0.001**
Cardiac death	6 (1.3)	24 (5.1)	**0.001**
Recurrent MI	3 (0.6)	8 (1.70)	0.130
Stroke	6 (1.3)	12 (2.6)	0.155
TLR	49 (10.4)	56 (11.9)	0.476
TVR	61 (13.0)	65 (13.8)	0.711
Any revascularization	80 (17.1)	96 (20.4)	0.186
All-cause mortality + MI + stroke	27 (5.8)	65 (13.8)	**<0.001**
Cardiac mortality + MI + TVR	68 (14.5)	91 (19.4)	**0.047**
Composite of MACE	102 (21.7)	146 (31.1)	**0.001**

MACE, major adverse cardiac event; MI, myocardial infarction; TLR, target lesion revascularization; TVR, target vessel revascularization.

Bold values indicates *p* < 0.05.

Based on an analysis of the study population, the high galectin-3 level showed a significant association with the composite of all-cause mortality, non-fatal MI, and stroke during long-term follow-up (unadjusted HR 2.570, 95% CI 1.640–4.027, *p* < 0.001), and the multivariate analysis, which included many variables listed in the statistical method, showed that the high galectin-3 level was associated with all-cause mortality, non-fatal MI, and stroke (adjusted HR 1.670, 95% CI 1.037–2.751, *p* = 0.044) (Table 3).

**Table 3:** Predictors of the composite of all-cause mortality, AMI, and stroke by univariate and multivariate Cox regression analyses.

	Unadjusted HR (95% CI)	*p*-value	Adjusted HR (95% CI)	*p*-value
Galectin-3 (binary)^a^	2.570 (1.640–4.027)	**<0** **.** **001**	1.670 (1,014–2.751)	**0**.**044**
Galectin-3 (continuous)^b^	1.052 (1.041–1.064)	**<0**.**001**	1.037 (1.023–1.052)	**<0**.**001**
Galectin-3 (log)^c^	3.647 (2.664–4.993)	**<0**.**001**	2.421 (1.652–3.548)	**<0**.**001**
Age	1.063 (1.042–1.085)	**<0**.**001**	1.047 (1.024–1.070)	**<0**.**001**
Male gander	1.304 (0.852–1.998)	0.222		
Hypertension	2.433 (1.482–3.993)	**<0**.**001**	1,966 (1.148–3.369)	**<0**.**001**
Diabetes	1.478 (0.982–2.224)	0.061		
Current smoking	0.820 (0.495–1.360)	0.442		
AMI as presentation	2.341 (1.553–3.526)	**<0**.**001**	1.85 (1.124–3.162)	**0**.**016**
LVEF	0.983 (0.974–0.992)	**<0**.**001**	0.986 (0.975–0.996)	**0**.**010**
Glucose	1.004 (1.001–1.006)	**0**.**003**	1.000 (0.997–1.003)	0.799
Hs-CRP	1.105 (1.076–1.135)	**<0**.**001**	1.071 (1.024–1.120)	**0**.**003**
eGFR	0.978 (0.970–0.987)	**<0**.**001**	0.991 (0.981–1.002)	0.095
CK-MB	1.002 (0.998–1.007)	0.255		
Trop-T	1.017 (1.051–1.028)	**0**.**003**	0.999 (0.983–1.015)	0.865
LDL-cholesterol	0.996 (0.991–1.001)	0.116		
HDL-cholesterol	0.999 (0.983–1.015)	0.875		
Triglyceride	0.996 (0.993–0.999)	**0**.**010**	1.000 (0.99–1.003)	0.841
Mean stent diameter	0.783 (0.510–1.203)	0.264		
Diseased vessel number(1)^d^	1.632 (0.990–2.693)	0.055	1.218 (0.728–2.037)	0.452
Diseased vessel number(2)^e^	1.994 (1.190–3.341)	**0**.**009**	1.086(0.630–1.871)	0.767

AMI, acute myocardial infarction; CI, confidence interval; CK-MB, creatine kinase-MB fraction; eGFR, estimated glomerular filtration rate; HDL, high-density lipoprotein; HR, hazard ratios; Hs-CRP, high-sensitivity C-reactive protein; LDL, low-density lipoprotein; LVEF, left ventricular ejection.

^a^ Categorical variable of galectin-3 (low galectin-3 vs. high galectin-3).

^b^ Continuous variable serum galectin-3 level.

^c^ Logarithm variable of serum galectin-3.

^d^ One-vessel disease (reference) vs. two-vessel diseases.

^e^ One-vessel disease (reference) vs. three-vessel diseases.

Bold values indicates *p* < 0.05.

We also showed the association of Gal-3 with the composite of all-cause mortality, non-fatal MI, and stroke according to whether ACS was present or not (Table 4). High Gal-3 levels were significantly associated with the composite of all-cause mortality, non-fatal MI, and stroke during long-term follow-up (unadjusted HR 2.812, 95% CI 1.580–5.003, *p* < 0.0001) in patients with ACS, but there was no such association (unadjusted HR 1.591, 95% CI 0.734–3.449, *p* = 0.240) in patients with non-ACS (*p* for interaction < 0.0001). Based on the analysis of the ACS study group, the clinical model included in the multivariate analysis showed that high Gal-3 levels were associated with the composite of all-cause mortality, non-fatal MI, and stroke (adjusted HR 2.178, 95% CI 1.200–3.951, *p* = 0.010), and high Gal-3 had a significant association in multivariate analysis including clinical model plus hs-CRP (HR 1.988, 95% CI 1.063–3.720, and *p* = 0.032).

**Table 4:** Association between galectin-3 and composite of all-cause mortality, non-fatal MI, and stroke according to the presentation of ACS.

	Non-ACS			ACS		
	Low Gal-3	High Gal-3	*p*-value	Low Gal-3	High Gal-3	*p*-value
Events, *n* (%)	12 (4.5)	14 (7.2)	0.218	15 (7.4)	51 (18.5)	<0.001
	HR	95% CI	*p*-value	HR	95% CI	*p*-value
Univariated						
Galectin-3 (binary)	1.591	0.734–3.449	0.240	2.812	1.580–5.003	<0.001
Multivariated^a^						
Galectin-3 (binary)^b^	1.374	0.598–3.217	0.480	1.988	1.063–3.720	0.032
Galectin-3 (continuous)^c^	1.036	1.001–1.017	0.012	1.048	1.030–1.066	<0.001
Galectin-3 (log)^d^	1.958	0.962–3.985	0.064	2.923	1.854–4.606	<0.001

ACS, acute coronary syndrome; AMI, acute myocardial infarction; CI, confidence interval; CK-MB, creatine kinase-MB fraction; eGFR, estimated glomerular filtration rate; Gal 3, galectin-3; HDL, high-density lipoprotein; HR, hazard ratios; LDL, low-density lipoprotein; LVEF, left ventricular ejection.

^a^ Adjusted by hs-CRP and clinical model including age, gender, diabetes, hypertension, current smoking, and hypercholesterolemia.

^b^ Categorical variable of galectin-3 (low galectin-3 vs. high galectin-3).

^c^ Continuous variable of serum galectin-3 level.

^d^ Logarithm variable of serum galectin-3 level.

**Figure 1:**
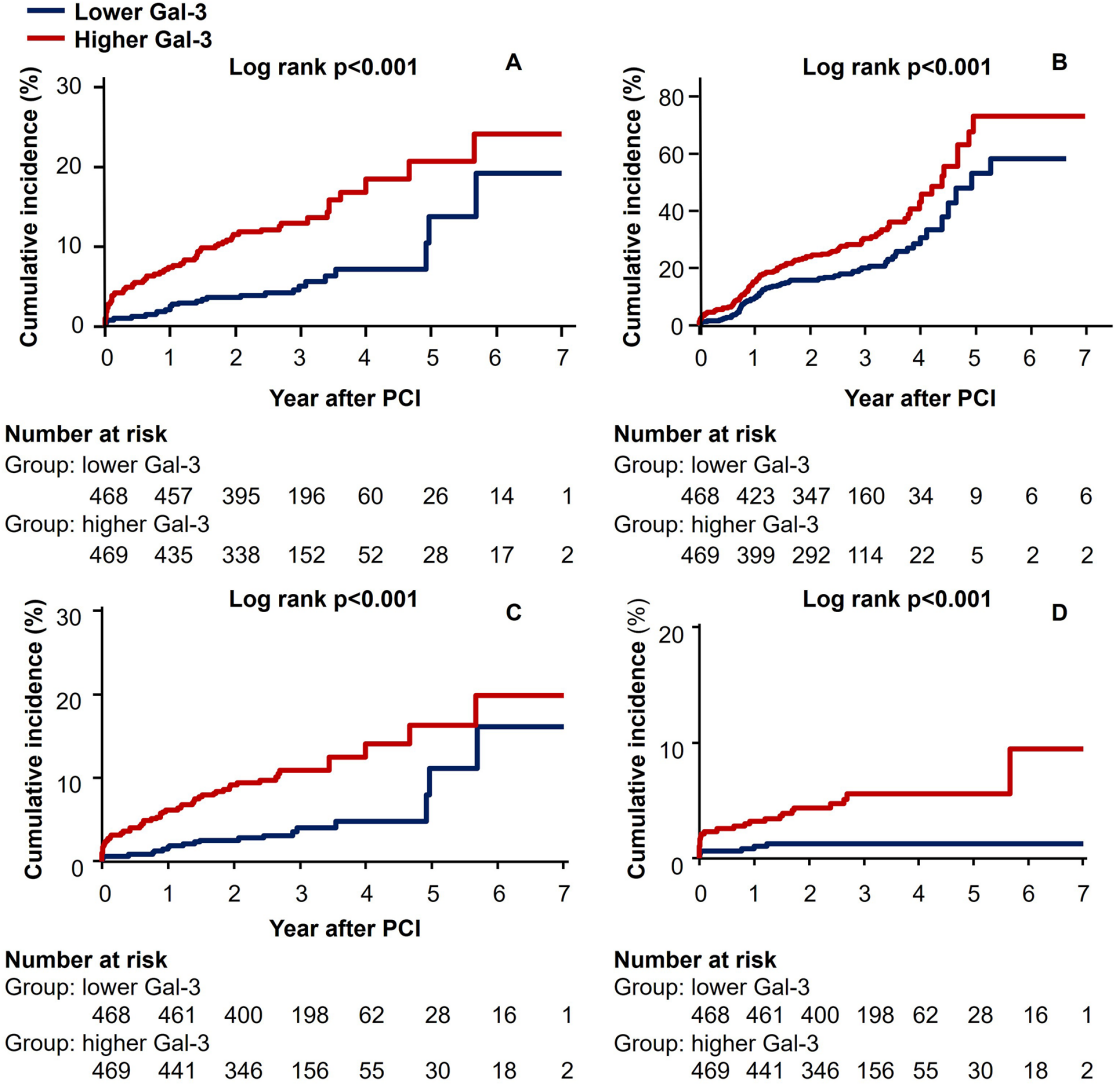
Kaplan–Meier curves for **(A)** composite of all-cause mortality, non-fatal MI, and stroke **(B)** composite of MACE, **(C)** all-cause mortality, and **(D)** cardiac mortality.

The Kaplan–Meier survival curves (Figure 1) showed that high galectin-3 showed significantly worse hard outcomes than the low galectin-3 as determined by the log-rank test; composite of all-cause mortality, non-fatal MI, and stroke (*p* < 0.001), all-cause mortality (*p* < 0.001), cardiac mortality (*p* = 0.001), and composite of MACE (*p* < 0.001).

**Figure 2:**
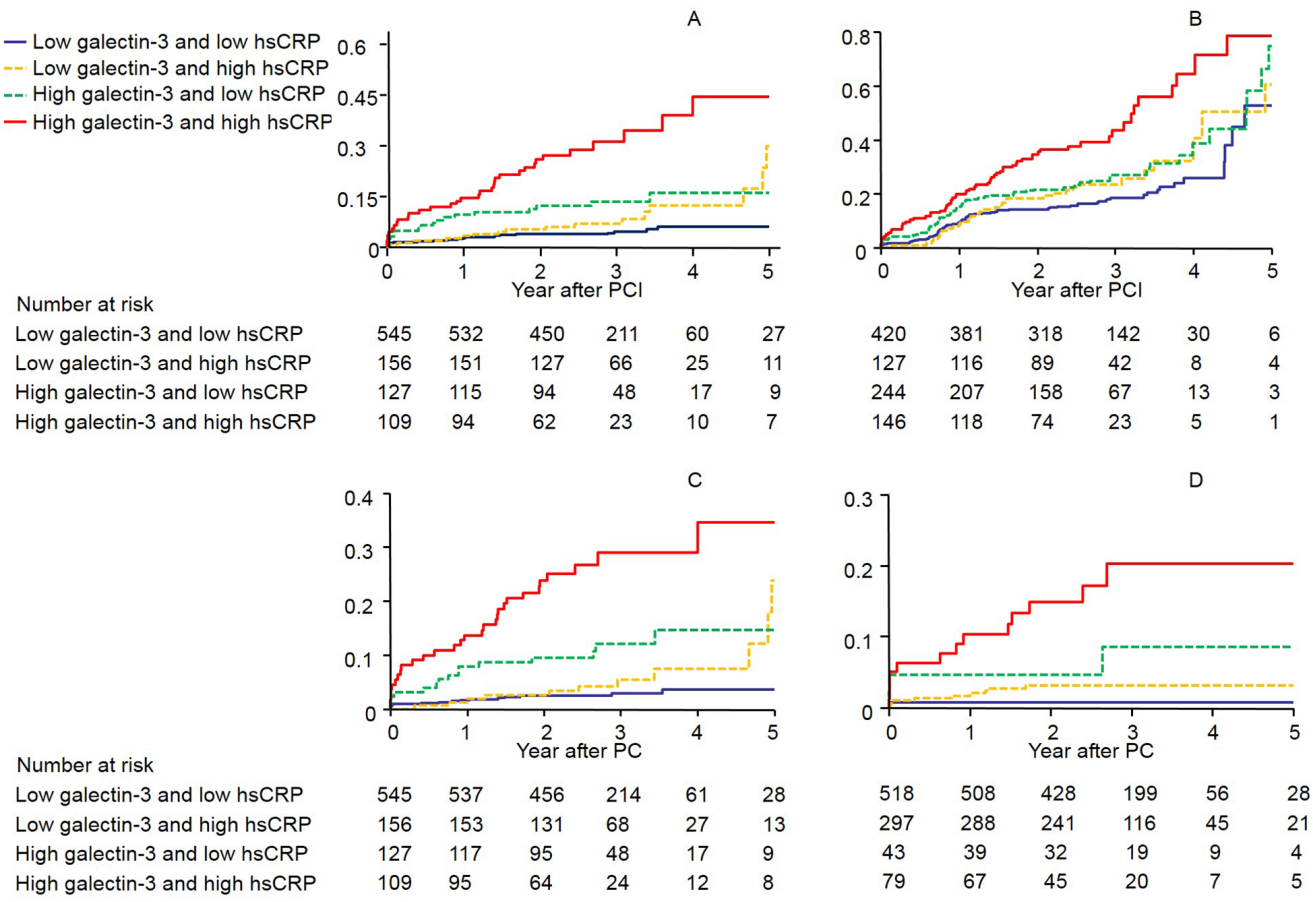
The Kaplan–Meier survival curves showing the rates of **(A)** composite of all-cause mortality, non-fatal MI, and stroke **(B)** composite of MACE, **(C)** all-cause mortality, and **(D)** cardiac mortality by galectin-3 and high-sensitivity C-reactive protein levels above or below the predefined cutoff values (log-rank *p* < 0.0001 for all events). CI, Confidence interval; CRP, C-reactive protein; HR, hazard ratios.

The serum galectin-3 cutoff values providing the best sensitivity and specificity for the prediction of MACE, all-cause mortality, and cardiac mortality were 10.41 ng/mL [area under the curve (AUC), 0.586; 95% CI 0.554–0.618; *p* = 0.0001], 12.71 ng/mL (AUC, 0.713; 95% CI 0.682–0.741; *p* < 0.0001), and 17.04 ng/mL (AUC, 0.755; 95% CI 0.726–0.782; *p* < 0.0001), respectively. In comparison with serum galectin-3, hs-CRP levels had a similar AUC (Table S2). In addition, galectin-3 and hs-CRP levels were slightly relatively correlated (r = 0.266, *p* < 0.0001, Figure S1), so each marker could detect different high-risk groups. We therefore constructed Kaplan–Meier survival curves after dividing the patients into four groups on the basis of whether they were above or below the cutoff values of galectin-3 and hs-CRP (Figure 2 and Table S3 show the adjusted HRs for each of the endpoints). Table S4 shows the adjusted HRs according to whether ACS was present or not.

### Subgroup analysis

**Figure 3:**
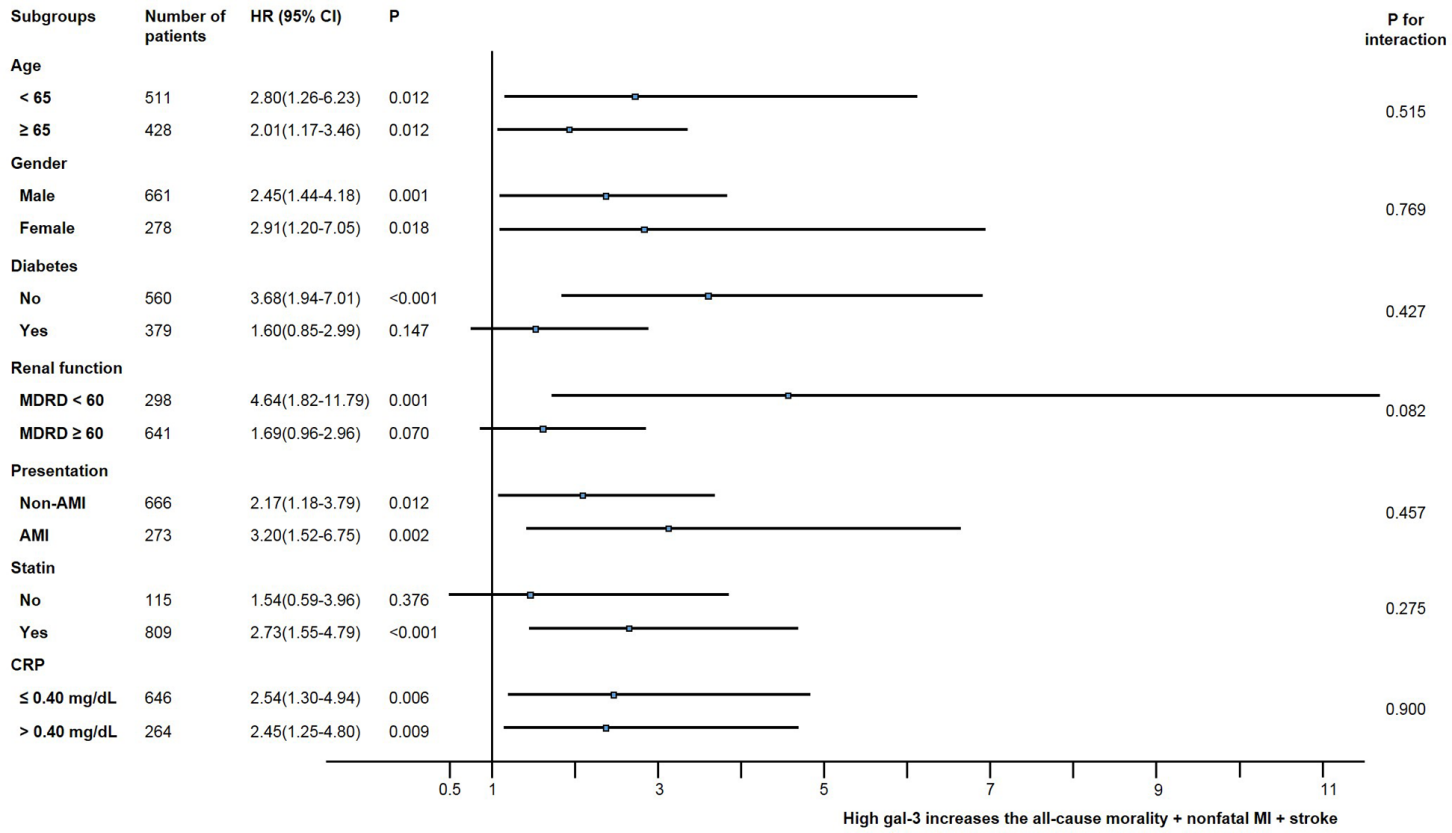
Comparative unadjusted hazard ratios of MACE for subgroups. ACS, acute coronary syndrome; CI, confidence interval; CRP, C-reactive protein; HR, hazard ratio; MDRD, modification of diet in renal disease, mL/min/1.73 m^2^.

We calculated the unadjusted hazard ratio for the composite of all-cause mortality, non-fatal MI, and stroke in various subgroups (Figure 3). The incidence of the composite of all-cause mortality, non-fatal MI, and stroke was numerically higher in the high galectin-3 group than in the low galectin-3 group in all subgroups, although statistical significance was not found in patients with diabetes, a relatively preserved renal function, and non-users of statin. There were no significant interactions between the galectin-3 level and the composite of all-cause mortality, non-fatal MI, and stroke among the seven subgroups.

### Incremental prognostic value of galectin-3 over the conventional clinical risk model

Table 5 shows the results of analysis using calibration, discrimination, and reclassification to evaluate the incremental usefulness of serum galectin-3 and hs-CRP level over the conventional clinical risk model for the prediction of all-cause mortality.

**Table 5:** Discrimination, calibration, and reclassification after the addition of galectin-3 and hs-CRP to the conventional clinical risk model on the composite of all-cause mortality, non-fatal MI, and stroke.

	Clinical model^a^ (reference)	Clinical model + Gal-3	Clinical model + hs-CRP	Clinical model + Gal-3 + hs-CRP
Hosmer–Lemeshow test, *p*	0.895	0.231	0.540	0.202
AIC	481.86	427.93	464.16	423.48
C-statistic (95% CI)	0.694 (0.625 to 0.762)	0.786 (0.723 to 0.848)	0.737 (0.670 to 0.803)	0.804 (0.743 to 0.864)
Difference in C-statistic		0.092	0.043	0.110
*p*-value		0.0009	0.0044	0.0002
IDI		0.064 (0.024 to 0.126)	0.041 (0.018 to 0.088)	0.089 (0.039 to 0.163)
*p*-value		<0.01	<0.01	<0.01
NRI continuous		0.297 (0.066 to 0.409)	0.209(−0.033 to 0.339)	0.360 (0.138 to 0.485)
*p*-value		<0.01	0.08	<0.01
Median improvement		0.009 (0.000 to 0.034)	0.000(−0.007 to 0.009)	0.014 (0.001 to 0.080)
*p*-value		0.03	0.88	0.02

AIC, Akaike information criterion; hs-CRP, high-sensitivity C-reactive protein; Gal-3, galectin-3; IDI, integrated discrimination improvement; NRI, net reclassification improvement.

^a^ Including age, gender, diabetes, hypertension, current smoking, and hypercholesterolemia.

The *p-*values for the Hosmer–Lemeshow statistics were 0.895 for the conventional clinical risk model, 0.231 for the addition of galectin-3, and 0.202 for the addition of galectin-3 and hs-CRP, signifying that these models were well calibrated. The Akaike information criterion was lower in the model containing galectin-3 or hs-CRP than in the conventional clinical risk model. Additional inclusion of galectin-3 improved the predictive power of the conventional clinical risk model including age, gender, diabetes, hypertension, diabetes, and hypercholesterolemia. The C-statistic for predicting all-cause mortality increased from 0.694 to 0.786 (*p* for difference = 0.0009). The addition of hs-CRP also improved prognostic value, and the C-statistic increased from 0.694 to 0.737 (*p* for difference = 0.0044). These improvements of discrimination by galectin-3 were similar to that by hs-CRP (*p* = 0.067). The IDI of adding biomarkers to the conventional clinical risk model improvement significantly (IDI for galectin-3 = 0.064, *p* for improvement < 0.01; for hs-CR*P* = 0.041, *p* for improvement < 0.01; and for the combined galectin-3 and hs-CRP = 0.089, *p* for improvement < 0.01). The addition of galectin-3 to the conventional clinical risk model gave an NRI of 0.297 (95% CI 0.066–0.409), *p* < 0.01), the addition of hs-CRP provided an NRI of 0.209 (95% CI −0.033 to 0.339, *p* = 0.08), and the addition of both galectin-3 and hs-CRP yielded an NRI of 0.360 (95% CI 0.138–0.485, *p* < 0.01). Both IDI and NRI showed improvement in their values when hs-CRP and galectin-3 were added to the conventional clinical risk model, and the improvement was the greatest when the two biomarkers were added simultaneously (IDI 8.9%, NRI 36%).

## Discussion

This study provides evidence that the initial serum galectin-3 level can be used as an important risk factor for predicting MACE, especial all-cause mortality in patients undergoing PCI with DES. To our knowledge, this is the first study to report showing the incremental prognostic value of the baseline galectin-3 level over conventional clinical risk factors in patients who underwent PCI using real-world registry data. The predictive value of galectin-3 was comparable with hs-CRP. Serum galectin-3 level is a significant predictor even after all other risk factors, including hs-CRP and conventional clinical risk factors, have been considered.

Traditional risk factors such as age, gender, hypertension, diabetes, smoking, and dyslipidemia have been integrated into a statistical model for risk prediction of future cardiovascular events after PCI. However, these factors do not fully explain future cardiovascular events. Thus, it is still needed to find predictors of subsequent cardiovascular events. In the present study, the serum galectin-3 level shows an incremental predictive value using statistical methods including C-statistics, IDI, and NRI. In contrast, the baseline hs-CRP level does not show an additional effect in the reclassification analysis. Our study shows that serum galectin-3 level can be a novel marker of cardiovascular risk in patients undergoing PCI because galectin-3 has a strong association with clinical outcomes in a multivariate model and provides incremental prognostic value (15).

Several studies have shown that galectin-3 is associated with the severity of heart failure, and it has important prognostic information in patients with heart failure (16, 17). Galectin-3 could be used as an additional tool for diagnosis and severity assessment of atherosclerotic disease in patients with suspected coronary artery disease (18). In a study on the clinical significance of Gal-3 in coronary artery disease, it was reported that it could be used as an additional tool for diagnosis and severity assessment of stable obstructive coronary artery disease and could help identify high-risk patients (19). Another study analyzed the utility of galectin-3 in patients with acute myocardial infarction and reported that elevated galectin-3 is significantly associated with remodeling, cardiac function, and clinical outcomes (20–22). Another study showed that patients with unstable angina had a higher level of galectin-3 than patients with stable angina, and a trend in correlation between serum galectin-3 levels and number of vessel disease in patients with CAD (23). A recent prospective cohort study revealed that galectin-3 is a strong independent predictor of cardiovascular mortality in high-risk patients referred for coronary angiography (24). In this study, the AUC value of galectin-3 for predicting cardiac events was relatively good at around 0.7, but when revascularization was included, it was relatively low at 0.586. There was no content on revascularization in many galectin-3 literature studies, but when only revascularization was looked at in this study, the utility of galectin-3 did not seem to be impressive. Our study is the first study to report the clinical impact of serum galectin-3 level in patients with CAD who underwent PCI with DES.

Inflammation and oxidative stress play a crucial role in all stages of atherosclerosis and vascular remodeling. Galectin-3 has been shown to be an important contributor to inflammation. Galectin-3 is colocalized with macrophages within atherosclerotic plaques, and its expression reflects the degree of plaque inflammation (25, 26). In our study, Gal-3 is able to predict cardiovascular events with more statistical significance in patients with ACS than in those with non-ACS when adjusted by clinical model plus hs-CRP, presumably because inflammation plays a larger role in ACS. The expression of Gal-3 was higher in unstable plaques than in stable regions from the same patient, and it affects endothelial cells, macrophages, and vascular smooth muscle cells, which are directly involved in the development of atherosclerosis, causing inflammatory plaques (27). However, the role of the circulating galectin-3 level for predicting the clinical outcomes of patients with CAD undergoing PCI with DES remains unclear; therefore, a further study for mechanical explanation is needed.

Our study has some limitations. First, our findings are subject to selection bias and confounding factors because the study included a registered population and was non-randomized with an observational design. Second, we measured galectin-3 only once at index PCI. It is not known whether galectin-3 levels fluctuate in plasma after PCI and follow-up. Third, coronary angiography was analyzed qualitatively and not quantitatively. A detailed quantitative coronary analysis may be helpful in further interpreting our findings. Finally, information on various factors that are associated with raised galectin-3 levels, such as fibrotic conditions of other organs, including the liver, lung, pancreatitis, and the kidneys, was not fully available to investigators.

## Conclusion

Galectin-3 is associated with cardiovascular events in patients undergoing PCI with DES. The serum galectin-3 level may serve as an independent predictor of cardiovascular events in patients with CAD undergoing PCI with DES.

## Data Availability

The raw data supporting the conclusions of this article will be made available by the authors without undue reservation.
