# Supplementary material for: Prognostic significance of serum galectin-3 in predicting cardiovascular outcomes after percutaneous coronary intervention with drug-eluting stents
Source: Front Cardiovasc Med. 2025 Jul 17;12:1563068. doi: 10.3389/fcvm.2025.1563068 (PMC12310497; doi:10.3389/fcvm.2025.1563068)
Supplement: Supplementary file 1 [file Datasheet1.pdf]

Supplementary Figure

Figure S1. Linear correlation of high sensitivity C-reactive protein levels with galectin-3 levels.

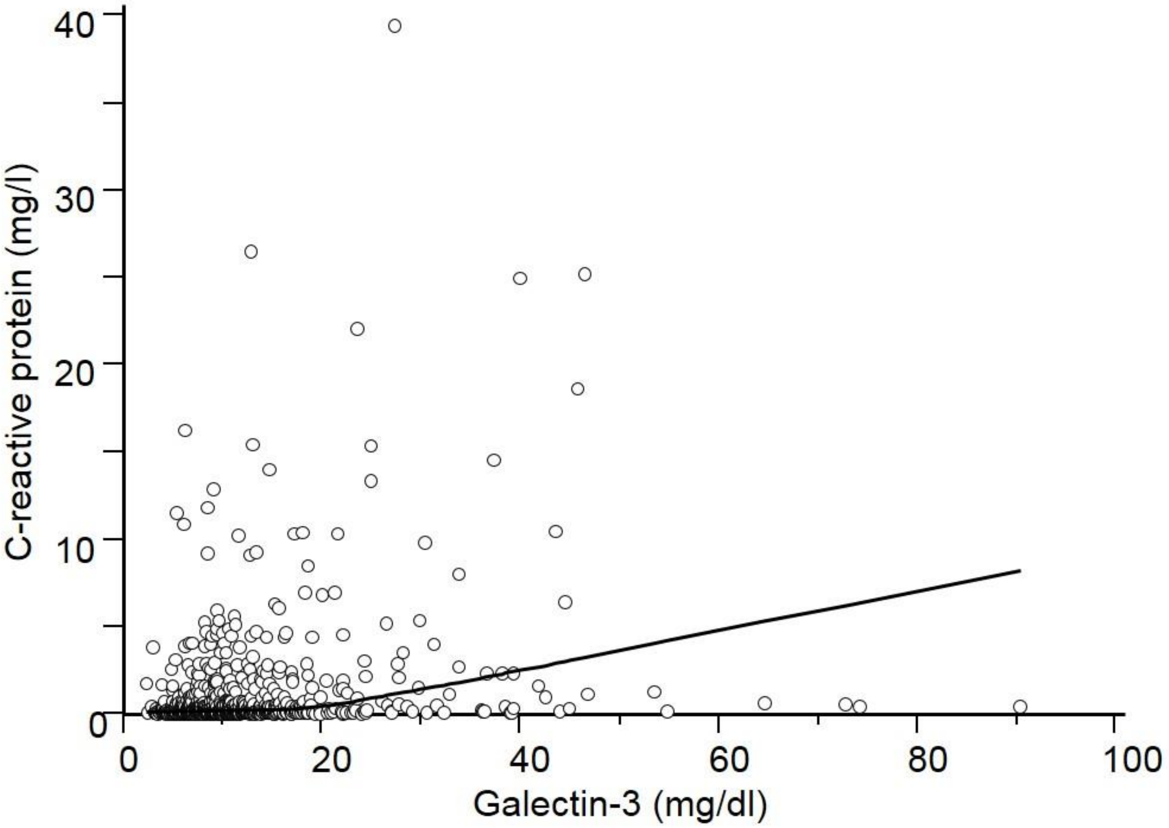

Supplementary Table

Table S1. Baseline demographic, clinical, and angiographic data ACS and Non-ACS

Variables	Non-ACS (n=459)	ACS (n=480)	p value
**Dermographics**			
Age, year	63.2 ± 9.4	62.5 ± 11.7	0.262
Age ≥ 65 yrs	209 (45.5)	219 (45.6)	0.978
Male gender	320 (69.7)	341 (71.0)	0.657
**Risk factors**			
BMI (Kg/m^2)	24.7 ± 3.8	23.9 ± 4.9	0.005
Diabetes mellitus	190 (41.4)	189 (39.4)	0.528
Hypertension	292 (63.6)	276 (57.5)	0.055
Current Smoking	73 (15.9)	141 (29.4)	<0.001
Family history of CAD	30 (6.5)	46 (9.6)	0.087
Prior history of stroke	36 (7.8)	27 (5.6)	0.174
Prior history of MI	30 (6.5)	31 (6.5)	0.962
Prior history of PCI	61 (13.3)	42 (8.8)	0.026
Prior history of CABG	7 (1.5)	5 (1.0)	0.510
**Medication prior PCI**			
Aspirin	263/412 (63.8)	126/370 (34.1)	<0.001
Statin	132/412 (32.0)	73/370 (19.7)	<0.001
**Discharge medication**			
Aspirin	453/456 (99.3)	467/469 (99.6)	0.683
Clopidogrel	459/459 (100)	479/480 (99.8)	1.000
Statin	389/456 (85.3)	420/468 (89.7)	0.041
Beta-blocker	355/466 (77.9)	352/468 (75.2)	0.344
ACEI or ARB	387/456 (84.9)	401/468 (85.7)	0.727
CCB	119/456 (26.1)	93/468 (19.9)	0.024
**Laboratory data**			
LVEF (%)	58.8 ± 16.3	53.0 ± 18.1	<0.001
Glucose (mg/dl)	115.1 ± 39.2	131.2 ± 59.8	<0.001
Creatinine (mg/dl)	1.16 ± 1.13	1.38 ± 1.67	0.016
eGFR, mL/min/1.73m^2	67.1 ± 18.3	66.3 ± 22.7	0.523
Reduced kidney function (eGFR < 60)	135 (29.4)	163 (34.0)	0.135
Hs-CRP (mg/l)	0.32 ± 0.71	1.64 ± 3.90	<0.001
CK-MB (ng/ml)	1.5 ± 1.9	21.3 ± 51.5	<0.001
Troponin-t (ng/ml)	0.3 ± 2.8	8.0 ± 15.5	<0.001
Total cholesterol (mg/dl)	170.5 ± 40.1	168.5 ± 47.3	0.493
Triglyceride (mg/dl)	145.1 ± 115.9	125.8 ± 91.0	0.005
HDL cholesterol (mg/dl)	43.9 ± 12.3	41.5 ± 13.2	0.005
LDL cholesterol (mg/dl)	99.3 ± 36.3	102.0 ± 39.5	0.277
Hypercholesterolemia	98 (21.4)	111 (23.1)	0.514
Lipoprotein (g/ml)	25.6 ± 25.4	24.1 ± 22.5	0.787
**Angiographic data**			
Left main disease	22 (4.8)	20 (4.2)	0.643
Disease vessels, n (%)			0.005
1 vessel	225 (49.0)	185 (38.5)	
2 vessels	130 (28.3)	170 (35.4)	
3 vessels	104 (22.7)	125 (26.0)	

Lesion Characteristics, n (%)			0.011
A/B1	70 (15.3)	104 (21.7)	
B2/C	389 (84.7)	376 (78.3)	
Stent number per patient	1.18 ± 0.53	1.15 ± 0.59	0.367
Mean stent diameter, mm	3.18 ± 0.41	3.17 ± 0.46	0.701
Total stent length, mm	28.15 ± 15.27	29.36 ± 16.17	0.239
Stent type			0.555
1st generation DES	380/452 (84.1)	385/466 (82.6)	
2nd generation DES	72/452 (15.9)	81 (17.4)	

Data are presented as mean ± SD or n (%).

ACEI/ARB=angiotensin converting enzyme inhibitor/angiotensin II receptor blocker; ACS=acute coronary syndrome; AMI=acute myocardial infarction; B2/C=complex lesion; BMI=body mass index; CABG=coronary artery bypass graft; CAD=coronary artery disease; CCB=calcium channel blocker; CK-MB=creatine kinase-MB fraction; DES=drug-eluting stents; eGFR=estimated glomerular filtration rate; Gal-3=galectin-3; HDL=high density lipoprotein, Hs-CRP=high-sensitivity C-reactive protein; LDL=low density lipoprotein; LVEF=left ventricular ejection; MI=myocardial infarction; NSTEMI=non ST segment elevation myocardial infarction; PCI=percutaneous coronary intervention; STEMI=ST segment elevation myocardial infarction

*1st generation DES=Cypher and Cypher Select (Cordis Corporation, Miami, FL, USA), Taxus Express and Taxus Liberté (Boston Scientific Corporation), and Endeavor (Medtronic Inc.); 2nd generation DES=Endeavor Resolute (Medtronic Inc.), XienceV, Xience Prime (Abbott Laboratories) and Promus, Promus Element (Boston Scientific Corporation)

Table S2. Optimal cutoff values for galectin-3 and hs-CRP with respect to cardiovascular events

		AUC	SE	95% CI	p	Sensitivity	Specificity	Criterion
All-cause mortality + MI + stroke	Gal-3	0.687	0.034	0.657-0.717	<0.0001	55.4	78.2	12.71
	Hs-CRP	0.694	0.030	0.663-0.724	<0.0001	56.2	73.9	0.4
MACE	Gal-3	0.586	0.022	0.554-0.618	0.0001	53.2	62.8	10.41
	Hs-CRP	0.593	0.021	0.561-0.625	<0.0001	38.3	75.3	0.39
All-cause mortality	Gal-3	0.713	0.038	0.682-0.741	<0.0001	62.5	78.0	12.71
	Hs-CRP	0.669	0.034	0.668-0.728	<0.0001	56.9	74.3	0.4
Cardiac mortality	Gal-3	0.755	0.057	0.726-0.782	<0.0001	56.7	88.6	17.04
	Hs-CRP	0.724	0.054	0.694-0.752	<0.0001	76.7	61.6	0.22

Gal-3=galectin-3; Hs-CRP=high-sensitivity C-reactive protein MACE=major adverse cardiac event; MI=myocardial infarction

Table S3. Risk of adverse outcomes by galectin-3 and hs-CRP levels above or below the predefined cutoff values.

	Events, n(%)	Model 1* HR	95% CI	p-value	Model 2** HR	95% CI	p-value
**All-cause mortality + nonfatal MI + stroke**							
Low Gal3-low CRP	25/546 (4.6)	1			1		
Low Gal3-high CRP	16/156 (10.3)	2.238	1.190~4.209	0.012	1.775	0.886~3.556	0.105
High Gal3-low CRP	17/127 (13.4)	2.531	1.353~4.734	0.004	2.684	1.397~5.154	0.003
High Gal3-high CRP	34/110 (30.9)	6.184	3.627~10.543	<0.001	3.457	1.772~6.746	<0.0001
p for trend	<0.001	<0.001			<0.001		
**MACE**							
Low Gal3-low CRP	82/421 (19.5)	1			1		
Low Gal3-high CRP	34/127 (26.8)	1.254	0.836~1.880	0.274	1.114	0.714~1.736	0.635
High Gal3-low CRP	69/244 (28.3)	1.385	0.994~1.932	0.055	1.461	1.030~2.071	0.033
High Gal3-high CRP	63/147 (42.9)	2.510	1.787~3.527	<0.0001	1.822	1.209~2.746	0.004
p for trend	<0.001	<0.001			0.017		
**All-cause mortality**							
Low Gal3-low CRP	16/546 (2.9)	1			1		
Low Gal3-high CRP	11/156 (7.1)	2.265	1.045~4.905	0.038	1.855	0.789~4.360	0.157
High Gal3-low CRP	15/127 (11.8)	3.409	1.665~6.979	0.001	3.959	1.839~8.521	<0.001
High Gal3-high CRP	30/110 (27.3)	8.142	4.345~15.257	<0.0001	4.488	2.034~9.900	<0.0001
p for trend	<0.001	<0.001			<0.001		
**Cardiac mortality**							
Low Gal3-low CRP	4/519 (0.8)	1			1		
Low Gal3-high CRP	9/298 (3.0)	3.744	1.147~12.217	0.029	4.236	0.865~20.754	0.075
High Gal3-low CRP	3/43 (7.0)	8.282	1.839~37.299	0.006	11.552	1.826~73.091	0.009
High Gal3-high CRP	14/79 (17.7)	19.727	6.410~60.715	<0.0001	17.406	3.387~89.439	0.0001
p for trend	<0.001	<0.001			0.001		

*Model 1: HRs have been adjusted for age, gender, diabetes, hypertension, current smoking, and hypercholesterolemia

**Model 2: HRs have been adjusted for Model 1 variables and additional covariates such as initial presentation of AMI, C-reactive protein, glucose, estimated glomerular filtration rate, left ventricular ejection fraction, troponin-T, triglyceride and lesion extent.

AMI=acute myocardial infarction; CI=Confidence interval; CRP=high sensitivity C-reactive protein; Gal-3=galectin-3; HR=Hazard ratios; MACE=Major adverse cardiac events

Table S4. The risk of the composite of all-cause mortality, nonfatal MI, and stroke by galectin-3 and hs-CRP levels above or below the predefined cutoff values.

	Events, n(%)	Model 1* HR	95% CI	p-value	Model 2** HR	95% CI	p-value
**Non-ACS patients**							
Low Gal3-low CRP	14/333 (4.2)	1			1		
Low Gal3-high CRP	4/55 (7.3)	1.768	0.576~5.427	0.319	1.290	0.309~5.386	0.727
High Gal3-low CRP	4/54 (7.4)	1.245	0.393~3.939	0.709	1.485	0.441~4.996	0.523
High Gal3-high CRP	4/17 (23.5)	3.785	1.120~12.795	0.032	3.195	0.607~16.826	0.171
p for trend	0.004	<0.001			0.560		
**ACS patients**							
Low Gal3-low CRP	11/213 (5.2)	1			1		
Low Gal3-high CRP	12/101 (11.9)	2.277	1.000~5.184	0.050	1.866	0.787~4.426	0.157
High Gal3-low CRP	13/73 (17.8)	3.310	1.470~7.456	0.004	3.811	1.642~8.843	0.002
High Gal3-high CRP	30/93 (32.3)	5.974	2.952~12.091	<0.0001	3.141	1.368~7.215	0.007
p for trend	<0.001	<0.001			0.009		

*Model 1: HRs have been adjusted for age, gender, diabetes, hypertension, current smoking, and hypercholesterolemia

**Model 2: HRs have been adjusted for Model 1 variables and additional covariates such as C-reactive protein, estimated glomerular filtration rate, left ventricular ejection fraction, and lesion extent.

AMI=acute myocardial infarction; CI=Confidence interval; CRP=high sensitivity C-reactive protein; Gal-3=galectin-3; HR=Hazard ratios; MACE=Major adverse cardiac events
